# Characterization and carbon mineralization of biochars produced from different animal manures and plant residues

**DOI:** 10.1038/s41598-020-57987-8

**Published:** 2020-01-22

**Authors:** Qamar Sarfaraz, Leandro Souza da Silva, Gerson Laerson Drescher, Mohsin Zafar, Fabiane Figueiredo Severo, Allan Kokkonen, Gustavo Dal Molin, Muhammad Izhar Shafi, Qudsia Shafique, Zakaria M. Solaiman

**Affiliations:** 1 Federal University of Santa Maria, 1000 Roraima Ave, Santa Maria, RS 97105-900 Brazil; 2 Department of Soil and Environmental Sciences, University of the Poonch Rawalakot, AJK, Pakistan; 3 Department of Soil and Environmental Sciences, University of Agriculture, Peshawar, KPK, Pakistan; 4 UWA School of Agriculture and Environment, and the UWA Institute of Agriculture, The University of Western Australia, Perth, 6009 Australia; 5 Department of Botany, Mirpur University of Science and Technology, AJK, Pakistan

**Keywords:** Environmental impact, Environmental sciences

## Abstract

Renewing carbon and re-establishing it again in the soil is one of the valuable means to cope with climate change. There are many technologies for carbon apprehension and storage, but the most important one gaining attention is biochar technology. So, to carbonize and return different biological materials back to the farmland, a comprehensive study was proposed to characterize and evaluate the carbon (C) mineralization of biochars produced from different animal manures and crop straws. Six types of biochars were prepared from animal manures (poultry litter, swine and cattle manures) and crop straws (rice, soybean, and corn straws). The biochars were analyzed for chemical characteristics (elemental variables, thermal decomposition, cation exchange capacity, pH, electrical conductivity, specific surface area, and surface functional groups) and an incubation experiment was conducted to evaluate C mineralization from soil biochar mixture. Biochars produced from crop straws resulted to have more C as compared to the biochars produced from animal manures. Concentration of nitrogen was low, while P, K, Ca, and Mg were found reasonably higher in all biochars except swine manure biochar. The plant-derived biochars presented lower CO_2_ emissions when incorporated to soil at 1 and 2% of C. Varying but all the biochars prepared represented an alkaline pH. Biochars prepared from the crop straws resulted to have more C, alkaline in nature, high CEC, low CO_2_ emissions, can sequester C and more suitable to enhance the soil fertility in comparison to biochars produced from other sources.

## Introduction

In the Southern region of Brazil can be found immense amounts of diverse animal manure and plant residues as the region have a massive number of animal farms and crop production. Swine and poultry farms are the principal sources of the manure produced in the region. Rice is cultivated on about 1,000 M ha area^1^, while soybean and corn are also major crops grown in the region (more than 6,000 M ha area)^2^. In the region, no-tillage planting system is used to grow crops for a long time, and crop straws are left on the surface at the time of harvesting. There may be noted an uneven decomposition of these organic materials and different environmental impacts can be noticed, such as releasing carbon dioxide (CO_2_) into the atmosphere and N can be immobilized by microorganisms or can be escaped into the atmosphere in the form of N_2_O, N_2_ and NH_3_.

Biochar is an alternative and beneficial strategy to dispose-off the animal manures and plant residues rather than keeping them on the place or applying to soil directly^3^. Biochar is a solid C rich material obtained from organic waste burning in the absence or low supply of oxygen^4^. A massive amount of different biomasses can be found to produce biochars such as animal manures, plant residues, sewage sludge, and agricultural wastes. Its production from different residues and wastes for the use of farm soils may be a valuable means to decrease the negative impacts of emissions (greenhouse gases) from the vast wastes, from manures and plant residues and also improving soil conditions by the addition C-rich biochars^5,6^. Biochar not only improves soil conditions but also be used efficiently as a promising new pollutant adsorbent in water and soil^7^.

There is several aspects which can influence the biochar production and characteristics such as the type of carbonization process, carbonization temperature^8^, time of carbonization, and type of feedstock. The biochars prepared at low temperatures have more yield, minor compactness of the aliphatic compounds and are amorphous. Amonette and Joseph reported minute CH_4_, H_2_ and C loss from biochars produced at low temperature^9^. Lang *et al*.^10^ found N losses from the biochars prepared above 400 °C. On the other hand, it is recommended that biochars produced at 450 °C are suitable for agriculture in relation to their production and nutrient concentrations^11^. As a consequence, biochars produced from different feedstock and from different techniques present assorted characteristics such as total carbon (TC)^12^, pH^13^, liming effect^14^, electrical conductivity (EC)^15^, cation exchange capacity (CEC)^13^, density (D), and specific surface area (BET-SSA)^16^.

The information above relates to the mode of preparation of biochars vary for its physical and chemical analysis concerning its use. A variety of processes used in different studies makes even more difficult in comparing the output regarding the influence of feedstock properties on the biochar characteristics. Keeping in mind the availability of the feedstock in the Southern region of Brazil, we decided to prepare biochars from poultry litter, swine and cattle manure, and rice, soybean, and corn straws. So, depending on the collection of feedstock type, we hypothesized that the biochars produced from animal manures and crop straws would be different in their chemical characteristics from each other so, can be differentiated and characterized for further use in agriculture.

The objectives of the study were to evaluate the characteristics of the biochars produced from different animal and crop residues and to evaluate the C mineralization, when biochars are incorporated to soil.

## Material and Methods

### Material collection

Animal manures (poultry litter, swine and cattle manures) were collected from the experimental areas of the Animal Science department and crop straws (rice and soybean straws) were collected from the experimental areas of the Soil Science Department at Federal University of Santa Maria (29°43′14.4′′S 53°43′31.2′′W) while corn straw was collected from a near municipality – Paraíso do Sul (29°35′10.3′′S 53°07′26.3′′W). Impurities (stones from manure and grasses from straws) from all raw materials were taken off manually. Raw materials were dried in the air-forced oven up to a constant mass at 60 °C. Manures were milled and passed through a mesh with 4 mm openings while all crop straws were milled and passed through a mesh with 8 mm openings.

### Biochar preparation

Biochars, swine manure biochar (SMB), poultry litter biochar (PLB), cattle manure biochar (CMB), rice straw biochar (RSB), soybean straw biochar (SSB), and corn straw biochar (CSB) were prepared at low temperature keeping in mind the findings of Novak *et al*.^17^ that biochars produced at low temperature reside more functional groups and are more suitable for agriculture use while biochars prepared at higher temperatures consume more energy as well as lose more nutrients. Prior to prepare biochars, all materials were well mixed to make a homogenized mixture. A known mass of raw material was kept in pre-weighed ceramic crucibles and put in a muffle furnace (Forno Jung, Number 7549, Brazil) at 450 °C with an increase in temperature 10 °C min^−1^ with the final residence time of 1 h. After 1 h of residence time in the muffle furnace, the furnace was turned off and was left down to cool at room temperature, then crucibles containing biochars were taken out and weighed. Biochar production was measured using formula as follows:

1 $${\rm{Biochar}}\,{\rm{production}}\,( \% )=\frac{{{\rm{Mass}}}_{({\rm{Biochar}})}({\rm{g}})}{{{\rm{Mass}}}_{({\rm{Feedstock}})}({\rm{g}})}\times 100$$

### Biochar analysis

To analyze, the biochars were milled and passed through a mesh containing 1 mm pore size. Total carbon (TC) and nitrogen (N) were measured in elemental analyzer (Thermo Scientific, Flash EA 1112, Milan, Italy). Triplicate samples of biochars were extracted by 0.1 M HNO_3_ solution following a method used at Embrapa^18^ to determine total P, K, Ca, and Mg. P was measured by using spectrophotometer following method Murphy & Riley^19^ while K, Ca, Mg and micronutrients were measured by the following method proposed by Tedesco *et al*.^20^.

Thermal decomposition analyses were performed to evaluate the decomposition of materials (biochars) by high heat (450 °C). Prior to evaluating decomposition, biochars were analyzed for moisture contents by putting a known mass of biochars in a muffle furnace at 105 °C for 24 h. Ash was measured by putting a known mass of biochars in small ceramic crucibles in a muffle furnace at 550 °C for 8 h, and ash content was calculated as follows.

2 $${\rm{Ash}}( \% )=\frac{{\rm{Mass}}\,{\rm{of}}\,{\rm{ash}}\,{\rm{after}}\,550\,^\circ {\rm{C}}\,({\rm{g}})}{{\rm{Mass}}\,{\rm{of}}\,{\rm{dry}}\,{\rm{biochar}}\,({\rm{g}})}\times 100$$

Fiber fractions of the biochars were also evaluated with duplicate samples, determining cellulose, hemicellulose, lignin and soluble fractions by the method of Van Soest *et al*.^21^ with some modifications in the procedure by increasing the burning time period of biochars in a muffle furnace at 550 °C for 10 h and lignin was evaluated.

Cation exchange capacity (CEC) was measured through the method used Enders *et al*.^22^ while electrical conductivity (EC) and pH were measured using distilled water with ratio 1:10 (w/v). Brunauer Emmett Teller specific surface area (BET-SSA) of biochars was measured at Ceramic Material Laboratory (LACER) of Federal University of Rio Grande do Sul, using Volumetric (Manometric) Gas Sorption method by Surface Area Analyzer (Quantachrome Instruments, N32-28E, USA)^23^. For Fourier-transform infrared (FTIR) spectroscopy, the biochar samples were mixed with spectroscopic-grade KBr and analyzed by Spectrometer Perkin-Elmer, Model Spectro One.

### Incubation experiment setup

The Typic Hapludult (U.S Soil Taxonomy) soil was collected from the experimental areas (29°43′14.2′′S 53°42′15.0′′W) of the Soil Science Department of Federal University of Santa Maria. Topsoil (0 to 20 cm) was collected, air-dried, ground and sieved. The texture of the soil was sandy loam (61.71% sand, 25.72% silt, 12.56% clay). Soil was analyzed from composite sample for pH_(1:2.5 w/v)_ (4.8), TC (1.2%), Ca (15.5 cmol_c_ dm^−3^), Mg (9.3 cmol_c_ dm^−3^) and Al (16.89 cmol_c_ dm^−3^). To evaluate the C mineralization, we set an experiment (80 g soil) in a biological oxygen demand (BOD) incubator at 25 °C for 49 days with 1 and 2% of C in accordance with carbon contents in biochar because of different densities of the biochar originated from animal manure and crop straw. One additional treatment (soil only) was included, instead of using individual control for each biochar type. Field capacity moisture content was maintained throughout the experimental period by adding water at each sampling day when needed.

### Statistical analysis

Results from each parameter were analyzed using software Statistix 8.1 and performed analysis of variance (ANOVA) to evaluate the difference among different biochars produced from the different feedstock. Least significance difference (LSD) was performed for parameters of biochars at 5% level of significance to differentiate the means. Data on the C mineralization was analyzed for ANOVA by software R version 3.5.1 with the assistance of RStudio, and the graphs were made using Sigmaplot.

## Results and Discussion

### Chemical properties of biochars

Chemical properties of biochars produced from different feedstocks at 450 °C pyrolyzing temperature for 1 h are presented in Table 1. Biochar yield was comparatively low as in previous studies^12^ because the oxygen was not controlled during the production of the biochars. Biochar production varied for all the materials showing maximum production in cattle manure and poultry litter (58% and 57%, respectively), while minimum production was noted by corn straw (26%).

**Table 1:** Chemical characteristics of biochars produced from swine manure (SMB), poultry litter (PLB), cattle manure (CMB), rice straw (RSB), soybean straw (SSB) and corn straw (CSB).

Nutrient	Animal Manures			Crop Straws			CV%
	SMB	PLB	CMB	RSB	SSB	CSB	
TC (%)	38.27 c	22.11 d	16.42 e	43.95 b	69.17 a	67.78 a	3.16
N (%)	3.00 a	1.82 b	0.95 c	0.87 c	2.13 b	0.79 c	11.66
P (%)	4.88 a	3.33 b	0.94 c	0.60 c	0.83 c	0.45 c	16.52
K (%)	3.67 c	5.60 b	2.66 d	5.97 a	0.69 f	2.23 e	4.30
Ca (%)	7.02 b	23.89 a	1.36 e	1.53 e	2.65 c	0.61 e	6.94
Mg (%)	5.84 a	2.79 b	0.07 c	0.05 c	0.13 c	0.04 c	7.55
Cu (mg kg^−1^)	20.7 b	7.7 d	31.2 a	17.6 c	20.5 b	18.4 c	5.71
Mn (mg kg^−1^)	462.6 b	262.7 c	476.6 b	1041.7 a	97.9 e	159.8 d	2.53
Zn (mg kg^−1^)	508.6 a	35.9 e	75.3 b	67.6 c	48.0 d	66.6 c	3.04
Fe (mg kg^−1^)	282.8 b	28.8 e	855.4 a	118.9 c	41.5 d	33.1 e	1.95

Different letters in horizontal lines show the significant difference among different biochars at 5% level of significance.

Maximum TC was found in SSB (69.17%) and CSB (67.78%) whereas TC in biochars produced from animal ranged from 16.42 to 38.27% in CMB and SMB, respectively. Less TC contents of the manure biochars can be attributed to the low pyrolysis temperature that did not allow to concentrate C in the feedstock. The high TC contents at biochars from soybean and corn straw might be related to the depletion of the H and O during the pyrolysis process^24^. Domingues *et al*.^25^ reported that the biochars produced from cattle manure have relatively low TC as compared to the plant materials because of the presence of more labile organic compounds in animal manure which can be lost rapidly at high temperature before the formation of recalcitrant compounds. Elevated TC can be connected to the C fixation in biomass at high temperature, that encourages the C sequestration. The results for TC of RSB are similar to the findings of the Jindo *et al*.^26^ who reported less TC (49%) contents in the biochar produced at low temperature (400 °C) from rice straw. N concentration is relatively low in all biochars. Relative low N concentration may be attributed to the high temperature during pyrolysis conditions, the burning of organic material, and the nitrogen loss as volatiles (NH_3_, N_2_O, NO_2_) from the original material. Lang *et al*.^10^ reported that the biochars produced above 400 °C contain relatively low N contents due to the loss of N from the parent material at high heat. Maximum N was determined in SMB (3%) following that found in PLB (1.82%). On the other hand, CSB (0.79%) showed the minimum concentration of N following the RSB (0.87%). The N concentration in PLB was similar to the N concentration (2%) of biochar produced by Chan *et al*.^27^ at 450 °C from poultry litter. Usually, legumes have more N concentration in plant tissues, and the biochar properties are in direct proportion to its original N concentration in the parent material. Moreover, Song and Guo^8^ prepared biochars at 300 to 600 °C with 50 °C interval for each biochar from poultry litter. Their results showed that biochars prepared at a lower temperature (i.e. 300 °C) had more nutrients concentration as compared to the biochars produced at 600 °C.

The P concentration in SMB (4.88%) and PLB (3.33%) is relatively high which is far higher than results from previous studies, a 19.0 g kg^−1^ P was reported by Gunes *et al*.^28^ from poultry manure biochar produced at 300 °C for 2 h. On the other hand, crop straw biochars presented lower P content. Minimum P was found in CSB (0.45%), which shows that P in the biochars produced from manures are richer than those in the biochars produced from crop residues. Similar to the trace elements, it is a possibility to preserve P in biomass during the slow pyrolysis process. Higher P content such as 43.0 g kg^−1^ was observed when biochars were produced from poultry litter by fast pyrolysis at 450 °C^29^. High P concentration in SMB and PLB may be accredited to the C loss and forming P stable. Biochars produced at low temperatures have more soluble P that becomes insoluble at high temperature^30^. The K concentration varied in all biochars ranging from 0.69% (SSB) to 5.97% (RSB), low concentration of K in SSB may be accompanied to the reason that the soybean straw was collected a week later when the harvesting was done, contrary to soybean, rice straw was collected on the same day, at same time when harvesting was done. Potassium is a nonstructural component of plant tissue and therefore could quickly be released after harvesting, even more after some rainfall. The observation related to K concentration in biochars are contradictory in literature, Yu *et al*.^31^ reported K loss from wheat straw at different increasing temperatures, on the other side Chan and Xu^32^ found to increase in K contents with increasing temperature and reported that biochar from poultry litter might have K contents from 1.41 to 7.49%.

The Ca and Mg contents are much higher in PLB and SMB comparing to the other biochars. Maximum Ca was found in PLB (23.89%) which can be related to the experiment from where poultry litter was collected, in the experimental site poultry trays were a bit higher from ground, so it has a possibility that the litter we had received from that area would be a mixture with poultry feed as well as eggshell. In the biochars from straws, Ca in CSB was the minimum (0.61%) following RSB (1.53%) as compared to the SSB. In contradiction, in biochars from poultry litter and cattle manures prepared at 500 °C by Wang *et al*.^33^ and Cao and Harris^34^ respectively, had more Ca and Mg contents.

Comparatively, micronutrients were found higher in the biochars produced from animal manures than the contents found in biochars produced from crop straws. Comparing overall biochars, Mn concentrations were higher in all biochars ranging from 97 mg kg^−1^ to 1041.7 mg kg^−1^ in both SSB and RSB. This behavior can also be related to the soil reduction process in flooded rice fields, increasing Mn solubility and its uptake by rice plants, resulting in a higher Mn content in rice straw compared to other crop residues. The elevated concentrations of micronutrients in biochars are due to the mass loss, which resulted in concentrate micronutrients in biochar products. Hossain *et al*.^35^ found in his research that trace elements concentration increases with the increase in temperature, there is a relation to the temperature used and with mass loss. He *et al*.^36^ evaluated that the temperature higher than 350 °C favors the stability of trace elements due to the mass loss.

All biochars had a different ash percent, highest in CMB (77.32%), while minimum ash percentage (10.32%) was found in CSB (Table 2). In comparison to the crop residues, the animal manures had more ash contents. Moreover, RSB had more ash as compared to the SSB and CSB, which may be ascribed to the partial change caused by the organic and inorganic substances during pyrolysis conditions^22^. Hemicellulose is the difference between acid detergent fiber and neutral detergent fiber in Van Soest’s method. Maximum (85.95%) hemicellulose was found in SSB, whereas minimum (44.08%) was noted in PLB. A small amount of cellulose was found in all biochars showing a maximum in CSB (0.116%) and a minimum in CMB (0.004%). Overall hemicellulose and cellulose contents were lower in biochars prepared from animal manures than the biochars from crop straws, except for RSB, which had 77.03% and 0.011% of hemicellulose and cellulose contents, respectively. The degradation of organic material obtained from animal wastes and plant residues shows a huge difference in lignin contents. Crop residues showed higher lignin 0.68, 0.96 and 0.97% for RSB, SSB and CSB, respectively, while the minimum one in animal manures was found in PLB (0.24%). Virheijen *et al*.^37^ reported that the chemical composition of biochars produced is directly proportional to the chemical compositions of the feedstock. Cellulose and lignin have thermal degradation in range of 240–350 and 280 to 500 °C^38^ respectively, it can be concluded that the material with higher lignin will have higher C contents while a material with low lignin contents will provide lower C content^37^. From our values regarding C contents in biochars from animal manures, we can emphasize SMB has more C as compared to the PLB and CMB so that it will have more lignin contents in the feedstock as well as in biochar. In the same way, all biochars from crop residues show higher values for lignin and C contents, which shows the positive relationship between them. Comparing biochars from animal manures and plant residues, more soluble fractions were found in animal manures for SMB, PLB, and CMB (39.53, 55.67 and 17.84% respectively). In the case of plant residues, RSB had higher (22.32%) soluble fraction as compared to SSB and CSB. The higher soluble fractions might be attributed to the elevated CEC of the RSB and for having more exchangeable cations (H^+^, K^+^)^39^.

**Table 2:** Thermal decomposition parameters (characteristics) of biochars produced from swine manure (SMB), poultry litter (PLB), cattle manure (CMB), rice straw (RSB), soybean straw (SSB) and corn straw (CSB).

Characteristic	Animal Manures			Crop Straws			CV%
	SMB	PLB	CMB	RSB	SSB	CSB	
Ash (%)*	50.33 c	72.61 b	77.32 a	37.97 d	14.39 e	10.32 f	5.74
H-Cell (%)	59.88 d	44.08 e	81.93 b	77.03 c	85.98 a	83.30 ab	2.11
Cell (%)	0.037	0.009	0.004	0.011	0.160	0.116	—
Lig (%)	0.60 c	0.24 d	0.25 d	0.68 b	0.96 a	0.97 a	6.12
SF (%)	39.53 b	55.67 a	17.84 d	22.32 c	13.07 e	15.81 d	5.57
Cell:lig	0.06 c	0.4 d	0.04 d	0.02 e	0.18a	0.12 b	0.01
Ash (%)_(Residual)_	0.18 c	0.13 d	0.64 a	0.24 b	0.06 e	0.08 e	0.21

Different lower-case letters in horizontal lines show the significant difference among different biochars at 5% level of significance. *Ash was measured direct by putting biochars in a muffle furnace at 550 °C for 10 h, ash residual, H-Cell (hemicellulose), Cell (Cellulose), Lig (lignin), SF (Soluble Fractions) were determined through Acid Detergent Fiber (H2SO4) and Neutral Detergent Fiber.

The cation exchange capacity (CEC) is the capacity of biochar to adsorb cations. In biochars produced from animal manures and crop residues, CEC was found considerably higher, minimum 117.5 (cmol_c_ kg^−1^) in PLB and SMB had maximum (170 cmol_c_ kg^−1^) (Table 3). This characteristic reinforces the capacity of biochars to hold cations when applied to soil, as well as an important alternative to improve degraded or low fertile soils. Cation exchange capacity of biochar can be associated with the deprotonation of H^+^ from the surface of the mineral particles or organic molecules with variables charges, in consequences to the increase of pH to alkaline of biochar^40^. Furthermore, abiotic oxidation reaction and carboxylation on the surface of the biochars particulates may contribute to surface charge generation, literally increasing the CEC of biochars^41^.

**Table 3:** Cation exchange capacity, C:N and pH, EC and specific surface area (SSA) of biochars produced from swine manure (SMB), poultry litter (PLB), cattle manure (CMB), rice straw (RSB), soybean straw (SSB), and corn straw (CSB).

Characteristic	Animal Manures			Crop Straws			CV%
	SMB	PLB	CMB	RSB	SSB	CSB	
CEC_(cmolc kg_^−1^_)_	170.0 a	117.5 c	127.5 c	162.0 b	165.0 b	152.0 b	6.49
C:N	12.74 e	12.11 e	17.28 d	50.74 b	32.50 c	66.69 a	3.63
pH	10.24 b	9.99 b	9.59 d	10.41 a	9.46 d	10.08 c	0.83
EC_(mScm_^−1^_)_	4.08 b	9.56 a	3.60 c	9.42 a	0.75 d	3.85 c	2.90
SSA (m^2^/g)	12.357	12.959	7.041	4.619	3.610	4.235	—

Different lower-case letters in horizontal lines show the significant difference among different biochars at 5% level of significance. pH and EC (electrical conductivity) were measured in (1:10) biochar water ratio because of more volume of the biochars obtained from crop straws.

The C:N ratio is an essential property of organic material because of the fact that it has a direct impact on the decomposition of the residues and N cycling in soil. In the present study, (Table 3) C:N is higher in biochars from crop residues (32.50, 50.75 and 66.69 for SSB, RSB and CSB, respectively) as compared to the C:N of biochars produced animal manures (12.11 to 17.28 in PLB and CMB, respectively). The CEC:C was found the maximum (7.18) from the biochars produced from poultry litter while minimum (2.24) was in CSB.

Biochars were highly alkaline, with pH ranging from 9.46 to 10.41 in SSB and RSB, respectively. Biochars prepared at low temperatures generally are neutral to slightly alkaline^6,32^, but in our case, all biochars prepared from animal manures and crop straws had higher pH as compared to the reported previously, same results were observed by Revell *et al*.^29^. The pyrolysis process distils the volatile and acidic compounds producing the biooil or biogas, keeping alkaline components in biomass. The pH of PLB was also similar to the findings of Chan *et al*.^27^. Electrical conductivity (EC) is the concentrations of all soluble salts present in solution. In the present findings, EC of the biochars produced from animal manures and crop residues was also higher ranging from 0.75 to 9.56 mScm^−1^ at pyrolysis temperature 450 °C. Soybean straw biochar presented the lowest EC while in PLB there was more EC as compared to others. The pH and EC of biochars increase with the increase in temperature of pyrolysis^8^.

Previous studies reported that surface area and pore size are related to the pyrolysis temperature and biomass nature^42^. Specific surface area (Table 3) of the biochars prepared from animal manures are higher as compared to the biochars produced from crop straws. The SSB and PLB presented the SSA almost three to four times of the SSB and CSB can be attributed to the presence of functional groups inside the pores, which may contribute decrease in the SSA^43^. Usually, the biochars produced from wood material exhibit a larger specific surface area as compared to the non-woody material (grasses, straws etc).

**Figure 1:**
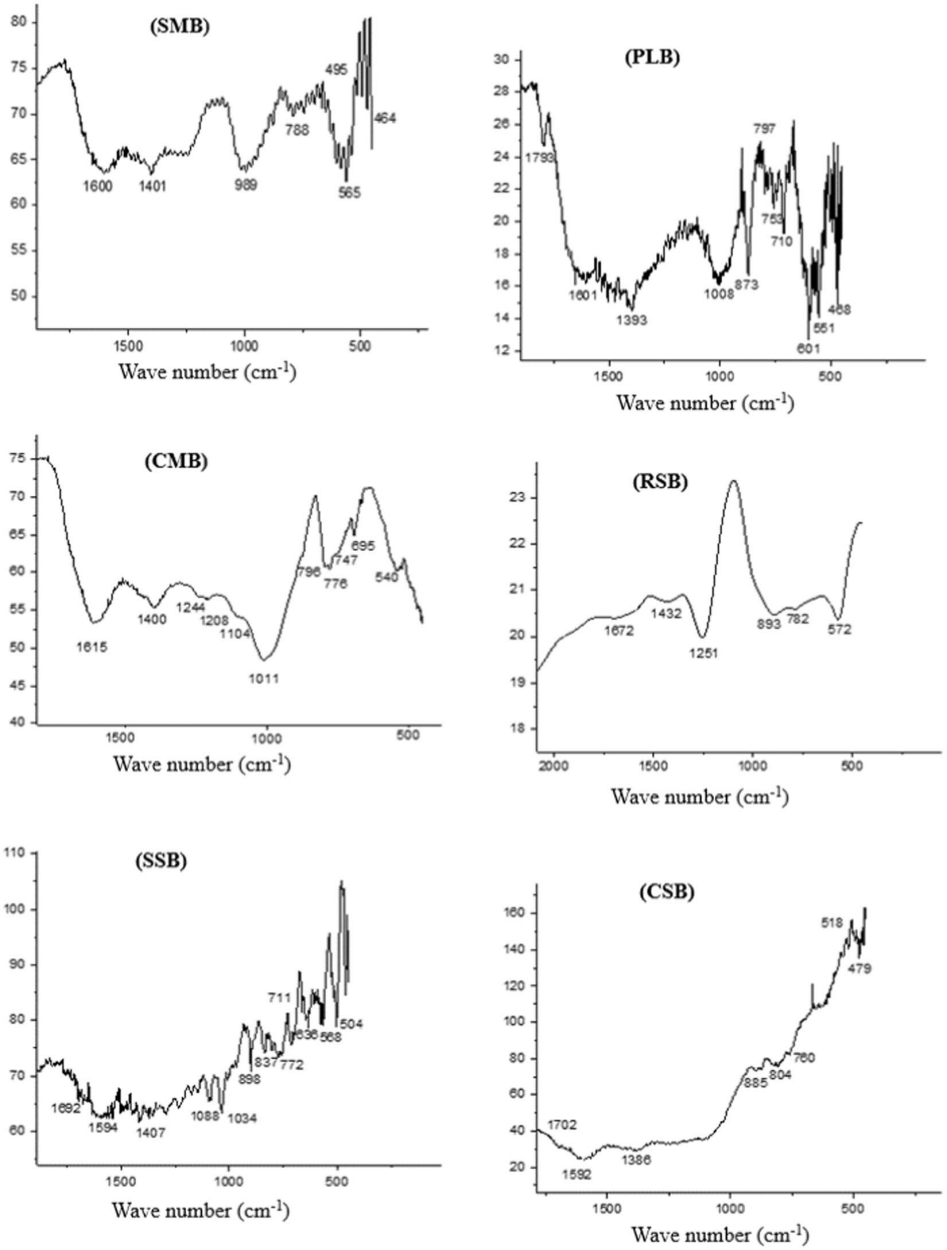
Fourier-Transform Infrared Spectroscopy (FTIR) of biochar produced from biochars produced from swine manure (SMB), poultry litter (PLB), cattle manure (CMB), rice straw (RSB), soybean straw (SSB), and corn straw (CSB).

Biochars produced from different animal manures, and crop straws were analyzed to evaluate the functional group’s structure of biochars. In the FTIR spectra (SMB1) from Fig. 1, peaks 1400 and 1600 cm^−1^ are related to the stretching O–H and C–O (Phenols)^44^. Peak 788 cm^−1^ illustrates the C=C aromatic rings while peaks smaller than 600 cm^−1^ are related to the vibrational chains of inorganic metals, e.g. M-X (M-metals and X-halogens for example peaks 565–495–464 cm^−1^ are related to the metal presence, maybe KCl and CaCl_2_). 989 cm^−1^: PO_4_^3−^ strains^45^. Peaks in spectra for PLB2 (Fig. 1), 1793 cm^−1^ related to the chains C=O (carboxyl, aldehyde, ketones and esters), 1601 cm^−1^ shows C=C, C=N and C=N bonds (aromatic components, acetone and quinone), 1393 cm^−1^ is related to the O–H bonding (phenols, ligneous syringyl), 1008 cm^−1^: P–O (symmetric and asymmetric stretching of PO_2_ and P(OH)_2_ in phosphate, 873 cm^−1^: C–H chains (aromatic C-H out of deformation plane, 797 cm^−1^: Pyridine (pyridine ring vibration and C–H deformation)^46^, 753–710 cm^−1^: C=C bonds with aromatic rings and spectra less than 601 cm^–1^ shows the presence of inorganic metals^45^. Figure 1 (CMB3), 1615: C=O or C=C chains (aromatic rings chains), 1400 cm^−1^: H–C–H (aliphatic compounds), 1104 cm^−1^: –C–O– chains, 1011 cm^−1^: PO_4_^3−^, 196–776 cm^−1^: Si–O–Si strains^47^. 1244 and 1208 cm^−1^: C=C aromatic rings and phenols respectively, 747–695 cm^−1^: C=C aromatic rings^45^. In spectra for RSB4 in Fig. 1, 1672 cm^−1^: strains C=C of aromatic rings, 1432 cm^−1^: O–H groups (carboxyl) and C–H, 1251 cm^−1^: C–O strain with characteristics of oxygenated functional groups (present in Cellulose), 893–782 cm^−1^: C– H with aromatic groups^48,49^, 572: inorganic metals like K and Ca. Spectra from Fig. 1 (SSB5), 1692–1594: hydroxyl chains (–COOH), 1088-898-837: the presence of –CO_3_^2−^, 1034: CH_2_ chains^50^, 772/1407: symmetric vibration chains (–COO–)^51^, 711: –C=C (aromatics rings). 636 and less: inorganic metal presence^45^. The CSB6 spectra from Fig. 1, 1592: C=O chains of aromatic rings, 1386: OH– strains (Phenols), 840: C–H chains of aromatic rings^52^, 1702: Carbonyl or carboxyl aromatic rings^53^, 885–760: C=C chains (aromatic rings). FTIR spectra for biochars clear that the moisture left the structure during the pyrolysis process, it may be notable the dehydration of C-OH groups and sharp division of some hydrocarbons object formed.

### Carbon dioxide (CO_2_) emission

**Figure 2:**
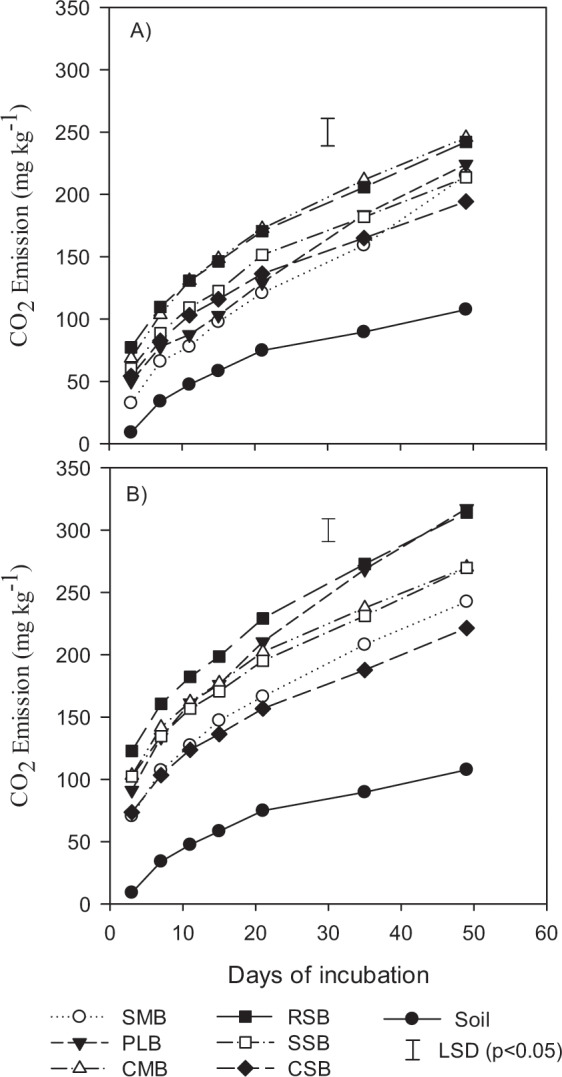
Carbon dioxide (CO_2_) emission from amended soil with biochars produced from swine manure (SMB), poultry litter (PLB), cattle manure (CMB), rice straw (RSB), soybean straw (SSB), and corn straw (CSB) at (**A**) 1% and (**B**) 2% dose of carbon.

Regardless of having added a considerable amount of TC (1 and 2%) in soil, lesser the amount of C mineralized from the treated soil (Fig. 2(A,B)). The efflux of CO_2_ remained changing during all collection periods, in the first temporal collection (3 days) of CO_2_ were the higher than the all other intervals, gradually decreased during the late period of incubation. The CO_2_ emission from biochars incorporated to soil at 1% C Fig. 2(A) shows that highest emission was observed in CMB (245.6 mg kg^−1^), following that 242.2 mg kg^−1^ from the treatment unit where RSB was applied. The same pattern was observed for PLB and RSB (Fig. 2B) which had maximum CO_2_ emission 317.35 mg kg^−1^ and 314.12 mg kg^−1^ respectively while the minimum was 221.4 mg kg^−1^ from the soil incorporated with CSB at 2%. Even with highest C contents in biochars, SSB and CSB emitted smaller amounts of CO_2_ that encourages the C sequestration. In overall comparison the CO_2_ emission results it can be found that the biochars produced from crop straws released less amount of CO_2_ as compared to the biochars from animal manures, which can be related to the more fixed C in the biochars produced from crop straws as compared to that of animal manures. Per day CO_2_ release was found higher 6.5 and 6.4 mg kg^−1^ in PLB and RSB when applied at 2% as compared to other biochars and control (2.2 mg kg^−1^). Shen *et al*.^54^ reported that in long period incubation periods addition of biochars decreases the CO_2_ efflux as compared to the short period incubation experiments, in contrast to their description, our results demonstrate the lower amounts of CO_2_ emissions when prepared at low temperature. Spokas and Reicosky also demonstrated that the biochars prepared at 400–510 °C could impede the C mineralization. The mechanism by which biochar addition to soil can affect the CO_2_ emission of soils is poorly stated^55^. Although effects of biochars addition to soil C cycling can impact the CO_2_ emissions in soil water gas system, the alteration in microbial biomass C that resulted from biochar additions consequently, may affect the C mineralization. The air-water balance established in the experiment and liming properties of biochars (pH > 9.5 for all biochars) could absorb the CO_2_ released from soil biochar system^56^.

Based on the biochars properties and nutrient concentrations, we found that the biochars produced from crop straws are rich in C contents which can sequester more C in soil in comparison with animal manures biochars having more CEC and pH, consequently on applying to an acidic soil such as Southern region of Brazil, can enhance soil fertility by increasing nutrients retention capacity and by increasing soil pH, keeping more nutrient available to crops.

## Conclusion

Biochars produced from animal manure, and crop straws using muffle furnace had demonstrated different characteristics, including the production of biochars percentage. Carbon and other nutrients concentration (P, K, Ca, Mg) were found in higher level except in the case of N, which was very low in all biochars. In thermal decomposition analysis of biochars, cellulose and lignin were decomposed readily by converting into soluble fractions whereas hemicellulose contents were partially decomposed during the pyrolysis process. Cation exchange capacity of biochars was higher which may increase the nutrient holding capacity of soil when fertilizer applied to soil. The pH and EC of the biochar were also higher, which assure that biochars prepared at 450 °C can play an essential role in increasing the pH of soil when applied to acidic soil. In mineralization of biochar, SSB and CSB emitted a negligible amount of CO_2_. Hence, on the bases of the findings of the study its can be concluded that the biochars produced at low temperature can sequester carbon instead of rapid C mineralization in soil.
