# Publisher Correction: Characterization and carbon mineralization of biochars produced from different animal manures and plant residues

**DOI:** 10.1038/s41598-021-88167-x

**Published:** 2021-04-13

**Authors:** Qamar Sarfaraz, Leandro Souza da Silva, Gerson Laerson Drescher, Mohsin Zafar, Fabiane Figueiredo Severo, Allan Kokkonen, Gustavo Dal Molin, Muhammad Izhar Shafi, Qudsia Shafique, Zakaria M. Solaiman

**Affiliations:** 1 Federal University of Santa Maria, 1000 Roraima Ave, Santa Maria, RS 97105-900 Brazil; 2 Department of Soil and Environmental Sciences, University of the Poonch, Rawalakot, AJK Pakistan; 3 Department of Soil and Environmental Sciences, University of Agriculture, Peshawar, KPK Pakistan; 4 UWA School of Agriculture and Environment, and the UWA Institute of Agriculture, The University of Western Australia, Perth, 6009 Australia; 5 Department of Botany, Mirpur University of Science and Technology, New Mirpur City, AJK Pakistan

Correction to: *Scientific Reports* https://doi.org/10.1038/s41598-020-57987-8, published online 22 January 2020

The original version of this Article contained a typographical error in the spelling of the authors Leandro Souza da Silva, Gerson Laerson Drescher, Fabiane Figueiredo Severo, Gustavo Dal Molin, Muhammad Izhar Shafi and Zakaria M. Solaiman which were incorrectly given as Leandro Silva, Gerson Drescher, Fabiane Severo, Gustavo Molin, Muhammad Shafi and Zakaria Solaiman. In addition, the Author Contributions section in this Article was incorrect.

“In this manuscript, Dr. Qamar is the corresponding author and the research was conducted by him along with colleagues from the Federal University of Santa Maria (Gerson Drescher, Fabiane Severo, Allan Kokkonen, Gustavo Molin) under the supervision of Prof. Leandro Silva. Ms. Qudsia Shafique and Muhammad Shafi helped in statistical analysis and as well as for interpretation of the results. Dr. Mohsin Zafar, Dr. Leandro Silva and Dr. Zakaria Solaiman being known scientists and researchers have done proof reading of the manuscript with respect to scientific terminologies and interpretation of the FTIR results.”

now reads:

“In this manuscript, Dr. Qamar is the corresponding author and the research was conducted by him along with colleagues from the Federal University of Santa Maria (Gerson Laerson Drescher, Fabiane Figueiredo Severo, Allan Kokkonen, Gustavo Dal Molin) under the supervision of Prof. Leandro Souza da Silva. Ms. Qudsia Shafique and Muhammad Izhar Shafi helped in statistical analysis and as well as for interpretation of the results. Dr. Mohsin Zafar, Dr. Leandro Souza da Silva and Dr. Zakaria M. Solaiman being known scientists and researchers have done proof reading of the manuscript with respect to scientific terminologies and interpretation of the FTIR results.”

The original Article has been corrected.

